# Laparoscopic Transperitoneal Nephrectomy in Non-functioning Severe Hydronephrotic Kidneys With or Without Renal Stone

**DOI:** 10.7759/cureus.3729

**Published:** 2018-12-13

**Authors:** Cem Yucel, Volkan Ulker, Erdem Kisa, Gokhan Koc, Yusuf O Ilbey

**Affiliations:** 1 Urology, University of Health Sciences Tepecik Training and Research Hospital, İzmir, TUR; 2 Urology, University of Health Sciences Tepecik Training and Research Hospital, Izmir, TUR

**Keywords:** non-functioning kidney, renal stone, laparoscopic nephrectomy

## Abstract

Aim

To investigate the effects of the additional presence of nephrolithiasis on the results of laparoscopic simple nephrectomy in patients with severe hydronephrotic non-functional kidneys.

Patients and methods

A total of 43 patients in whom severe hydronephrosis had been determined on spiral computerized tomography, who had a renal function of lower than 10% according to the dimercaptosuccinic acid (DMSA) renal scintigraphy, and who had undergone a laparoscopic simple nephrectomy due to persistent renal pain and/or recurrent urinary tract infection and/or unresolved hypertension with multidrug treatment, were included in the study. The patients were divided into two groups based on the presence of renal stone as those with stone formation (Group 1) and those without stone formation (Group 2). The groups were compared with regard to the patients’ demographic characteristics, operation durations, pre- and postoperative hemoglobulin and creatinine levels, percentage of change of postoperative hemoglobulin levels, complications, transfusion requirements, and durations of hospitalization.

Results

Overall, there were 43 patients including 19 patients in Group 1 and 24 patients in Group 2. Although the durations of operations, the durations of hospitalization, and the rates of change of hemoglobulin were higher in the patients in Group 1, these differences were not statistically significant. Postoperative complications were observed in 21 (48.8%) of the 43 patients. Postoperative complications were observed in 13 patients in Group 1 and in eight patients in Group 2. This difference was determined to be statistically significant (p<0.01).

Conclusions

We observed that except for the postoperative complication rates, the laparoscopic nephrectomy results in patients with severe hydronephrotic non-functional kidneys with or without stone were similar.

## Introduction

Today, minimally invasive surgery has become the standard treatment for many urological pathologies. Following the performing of laparoscopic nephrectomy for the first time in 1991 by Clayman et al. [1], this method has gained widespread recognition. Laparoscopic simple nephrectomy is performed in benign pathologies. Despite the word simple in its name, the increased perirenal adhesions secondary to infectious processes may render this surgery difficult. This surgery may become even more difficult due to limited space and potentially dense adhesions around the kidney, particularly in patients with hydronephrotic kidneys as opposed to patients with small kidneys. In addition to ureteropelvic junction (UPJ) obstructions, one of the most important factors in the developement of hydronephrotic kidney is longstanding renal stones that may lead to hydronephrosis and non-functioning kidneys [2]. In patients with kidney stone disease, laparoscopic nephrectomy may be complicated by peripheral adhesions developing secondary to previous episodes of pyelonephritis or pyelonephrosis. To our knowledge, there is no article in the literature investigating the effects of the additional presence of stone disease on the results of laparoscopic nephrectomy in patients with severe hydronephrotic non-functional kidney. Our study is the first article investigating this subject and it aimed to investigate the effects of the additional presence of nephrolithiasis on the results of laparoscopic nephrectomy in patients with severe hydronephrotic non-functional kidneys.

## Materials and methods

Patients and study design

The data of patients who had undergone a laparoscopic nephrectomy operation due to non-functional hydronephrotic renal disease in our institution between February 2012 and December 2017 were retrospectively reviewed. All patients underwent biochemical and radiological analysis before surgery. Samples for complete blood count, serum creatinine, platelet count, bleeding and coagulation profile and urine culture were obtained from all of the patients preoperatively. A total of 43 patients in whom severe hydronephrosis (grade 4) had been determined on the spiral computerized tomography, who had a renal function of lower than 10% according to the dimercaptosuccinic acid (DMSA) renal scintigraphy, and who had undergone a laparoscopic simple nephrectomy due to persistent renal pain and/or recurrent urinary tract infection and/or unresolved hypertension with multidrug treatment, were included in the study. Patients who had been operated with open surgery, those who had a history of previous abdominal surgery, those who had hydronephrosis milder than grade 4, those who had undergone nephrectomy due to renal mass or trauma, and those who had non-functioning kidneys were excluded from the study. All surgical operations had been performed through a transperitoneal approach. Consent from the ethics committee was not required because of the retrospective nature of this study. Written informed consent to undergo surgery was routinely obtained from each surgical patient.

The patients were divided into two groups based on the presence of renal stones as those with stone formation (Group 1) and those without stone formation (Group 2). The groups were compared with regard to the patients’ demographic characteristics, operation durations, pre- and postoperative hemoglobin and creatinine levels, percentage of change of postoperative hemoglobin levels, complications, transfusion requirements, and durations of hospitalization. The postoperative complications were evaluated using the modified Clavien system [3]. According to this classification, the complications were classified in five grades. Grade 1 comprises complications not requiring surgery or radiological intervention and those requiring antiemetic, antipyretic, analgesic, diuretic, electrolyte, and physiotherapy applications postoperatively. Grade 2 comprises complications requiring applications such as blood transfusions, parenteral nutrition, and antihypertensive drug treatments. Grade 3 consists of complications requiring surgery, an endoscopic or radiological intervention. Grade 4 comprises complications causing life-threatening situations such as organ failure. Grade 5 comprises complications resulting in death.

Surgical technique

Under general anaesthesia, all patients were positioned in the modified flank position with the side to the operated on top. Entry was made from the paraumbilical region with a Veress needle, and pneumoperitoneum was created by providing a pressure of 20 mmHg with carbon dioxide. Following insufflation, entry was made at the same region with a 12 mm trocar and the camera port was placed. The other two trocars were placed under direct vision. A 12 mm port was inserted close to the crista iliaca superior, 11 cm to the camera port, and a 10 mm port was also inserted at an 11 cm distance to the camera. In cases where the extraction was insufficient, a fourth trocar (5 mm) entry was made and better visualization of the surgical field was provided. Following installation of the trocars, the intraabdominal pressure was reduced to 12 mmHg. In left-sided surgeries, the Toldt line used in order to lower the descending colon was incised. In right-sided surgeries, however, the incision was made between the Toldt line and the colonic flexure. The gerota fascia was incised and the lower pole of the kidney was visualized. Subsequently, after having visualized the psoas muscle, the ureter was found and followed until the renal pelvis. The upper one-third portion of the ureter was clipped and cut. The renal artery and vein were reached. The renal vein and the renal artery were clipped with Hem-o-lock clips (Teleflex. NC, USA). The dissection was advanced towards the superior and the posterior of the kidney. Hemostasis control was performed under low pressure (6 mmHg). The excised kidney was removed from the abdominal cavity within an endo-bag.

Statistical analysis

The consistency of the numerical variables with the normal distribution was analyzed using the Shapiro-Wilk test. The categorical variables and numerical variables were presented as number (%) and mean ± SD, respectively. The relationship between two categorical variables was investigated using the Chi-square and the Fisher’s exact tests. The difference between two independent means was analyzed using the parametric Student t test or the non-parametric Mann-Whitney U test. Analysis was performed using the Statistical Package for the Social Sciences (SPSS Inc, Chicago, Illinois, USA version 22.0) and a p value of <0.05 was considered as significant.

## Results

Overall, there were 43 patients including 19 patients in Group 1 and 24 patients in Group 2. The average age of the patients was 46.1±16.3. Of these patients, 22 were male and 21 were female. While right nephrectomy was performed in 20 patients, left nephrectomy was performed in 23 patients. There was no statistically significant difference between the groups with regard to age, gender, the nephrectomy side, and the mean body mass index (BMI). The patients’ demographic data have been presented in Table 1. The mean stone density in Group 1 was 643±373 HU and the mean size of the stone was 24.7±8.8 mm. Three patients in Group 1 had a history of extracorporeal shock wave lithotripsy (ESWL), one had a history of pyelolithotomy and two had a history of ureterorenoscopy.

**Table 1:** Demographic Data of Patients BMI - body mass index

Variables	Overall (n=43)	Group 1 (n=19)	Group 2 (n=24)	p value
Mean age (years)	46.1±16.3	46.2±17.8	46.0±15.5	0.982
Gender (n, %)				
Male	22 (51.2)	8 (42.1)	14 (58.3)	0.290
Female	21 (48.8)	11 (57.9)	10 (41.7)	
Side (n, %)				
Right	20 (46.5)	7 (36.8)	13 (54.2)	0.258
Left	23 (53.5)	12 (63.2)	11 (45.8)	
Mean BMI (kg/m^2^)	25.7±1.4	25.4±1.3	26.1±1.7	0.826

Despite the operation durations, the durations of hospitalization, and the hemoglobin changes being higher in patients in Group 1, these differences were not statistically significant. The patients’ preoperative and postoperative data have been summarized in Table 2. All procedures were completed without the need for open conversion. Intraoperative bowel damage was observed in one patient in Group 1 and one patient in Group 2, and the general surgery doctors participated in the operation and the bowel damage was laparoscopically repaired. The postoperative complications were evaluated according to the Clavien classification system. Postoperative complications were observed in 21 (48.8%) of the 43 patients. Postoperative complications were observed in 13 patients in Group 1 and in eight patients in Group 2. This difference was determined to be statistically significant (p<0.01). Grade 2 (%23.2) complication was observed most frequently among all of the patients. Fortunately, no grade 4 or 5 Clavien complication was observed in any of the patients. Postoperative blood transfusion was administered to two (4.6%) patients in Group 1, and in four (9.3%) patients in Group 2. This difference was not statistically significant (p=0.38). One patient in Group 1 was followed up at the intensive care unit for two days due to poor general condition. Postoperative fever developed in one patient in Group 1 and in two patients in Group 2, and these patients’ symptoms abated following antibiotic treatment. Open abdominal exploration was performed on the first postoperative day in two patients in Group 1 due to severe decrease in hemoglobin. While a severely decreased hemoglobin level resulted from adrenal vein damage in one case, it resulted from blood leak from the renal pedicle due to sliding of the vascular clamp. A tube thoracostomy was performed in one patient in Group 1 upon development of postoperative severe pleural effusion. Clavien grade 3 complication was not observed in the patients in Group 2. Comparison of the postoperative complications has been demonstrated in Table 3.

**Table 2:** Preoperative and Postoperative Data of Patients

Variables	Overall (n=43)	Group 1 (n=19)	Group 2 (n=24)	p value
Operation time (min)	211±72	228±84	200±61	0.480
Length of hospital stay (days)	3.86±1.4	3.89±1.6	3.83±1.3	0.899
Preoperative hemoglobin (g/dL)	13.9±1.6	13.5±1.7	14.2±1.4	0.091
Postoperative hemoglobin (g/dL)	11.8±1.5	11.4±1.7	12.0±1.3	0.209
Hemoglobin change (%)	15.2±7.3	15.6±4.8	14.8±6.2	0.250
Preoperative creatinine (mg/dL)	1.0±0.3	1.0±0.3	1.1±0.4	0.976
Postoperative creatinine (mg/dL)	1.1±0.4	1.0±0.6	1.2±0.1	0.785
Drainage left (days)	2.9±1.6	2.8±1.2	3.1±1.5	0.885

**Table 3:** Comparison of the Postoperative Complications

Variables	Overall (n=43)	Group 1 (n=19)	Group 2 (n=24)	p value
Intraoperative complications (n, %)	2 (4.6)	1 (2.3)	1 (2.3)	1.0^b^
Requirement for transfusion (%)	6 (13.9)	2 (4.6)	4 (9.3)	0.38^b^
Postoperative complications (n, %)	21 (48.8)	13 (30.2)	8 (19.6)	<0.01^a^
Clavien classification				
grade 1	8 (18.6)	6 (13.9)	2 (4.6)	0.25^b^
grade 2	10 (23.2)	4 (9.3)	6 (13.9)	0.42^b^
grade 3	3 (6.9)	3 (6.9)	0	NA
grade 4	0	0	0	NA
grade 5	0	0	0	NA
^a^ Chi-square test; ^b^ Fisher’s exact test; NA, not applicable				

The mean stone density in Group 1 was 643±373 HU and the mean size of the stone was 24.7±8.8 mm. Three patients in Group 1 had a history of extracorporeal shock wave lithotripsy (ESWL), one had a history of pyelolithotomy, and two had a history of ureterorenoscopy.

## Discussion

In this study, we investigated the effect of the presence of kidney stones on the results of laparoscopic simple nephrectomy in patients with severe hydronephrotic non-functional kidneys and we concluded that the presence of postoperative complications was higher in hydronephrotic non-functional kidney disease patients who had kidney stones. In recent years, as a result of technological advances, minimally invasive methods have begun to replace open surgery. Owing to the shorter duration of hospitalization, the lower requirement for analgesics, better cosmetic results, and the short recovery period in laparoscopic nephrectomy operations compared to open surgery, it is known that a faster return to daily life is provided [4]. Today, with the increase in clinical experience, laparoscopic simple nephrectomy is preferred in non-functional kidney disease patients who have accompanying complaints of recurrent infection, severe lumbar pain or severe renovascular hypertension. Laparoscopic nephrectomy can be performed through a transperitoneal or a retroperitoneal approach. In our clinic, due to provision of better maneuverability as a result of the large space between the ports and due to our wider experience, the transperitoneal approach is preferred.

Despite the word simple, the simple laparoscopic nephrectomy procedure in patients with hydronephrotic non-functional kidneys may become a more difficult operation as opposed to the procedure in patients with non-functional small kidneys. In their study, Challacombe et al. compared the results of transperitoneal laparoscopic nephrectomy they performed on 11 small non-functional kidneys and 19 giant hydronephrotic non-functional kidneys and they observed a longer duration of operation and more blood loss in patients with hydronephrotic non-functional kidneys [5]. They had used the technique of puncturing and draining the kidney early in the dissection. We frequently use the same technique in hydronephrotic kidney patients for safer dissection of the renal artery and vein.

The most important factor in the etiology of hydronephrotic non-functional kidney is UPJ obstructions. Factors such as repeated infections observed in childhood or later stages of life, kidney stones, vesiculoureteral reflux, and pressure onto the UPJ from the outside have been blamed in the development of these obstructions [6]. Development of stenosis in UPJ may pave the way for development of infection of the urine collected in the pelvis and development of stones.

In the study of Kaba et al. where they compared the results of laparoscopic surgeries in 15 non-functional inflammatory kidney patients with stones and 19 patients without stones, they did not determine a significant difference between the groups with regard to surgical complications [3]. Similarly, in their study where they compared 27 patients with kidney stone who were of similar age and similar previous surgeries with another 27 patients with other benign pathologies, Tepeler et al. did not determine significant differences between the groups with regard to postoperative complications [7]. Contrary to these studies, in our study, the development of postoperative complication was observed to be statistically higher in the group with kidney stones. Different to the aforementioned studies, only the results of severely hydronephrotic patients had been included in our study. Despite the absence of a statistically significant difference between the Clavien grade 1 and 2 complications between the groups in our study, Clavien grade 3 complications were only observed in the group with kidney stones.

It is known that there is a strong relationship between urinary tract stones and urinary tract infections [8]. In our study, due to the densities of the stones in patients with kidney stones, we think that previously experienced infections have played an effective role in the development of these stones. Adhesions developing as a result of calculous pyelonephritis may render renal pedicle dissection difficult. Renal pedicle dissection may also be rendered difficult secondary to large pelvic stone pressure. Previous urinary tract infections may lead to severe adhesions, especially in the perinephritic region, and this may complicate laparoscopic surgery.

In their study where they published the results of 96 patients who had undergone simple laparoscopic nephrectomy due to stone disease, Angerri et al. had observed Clavien grade 3 complications in three patients due to the complications of spleen laceration, pleural injury, and bowel obstruction [9]. Furthermore, they had reported the conversion to open surgery in seven patients. In an another study on 17 cases undergoing laparoscopic nephrectomy with inflamed kidneys, blood transfusion was required in two cases, while the operation was converted to open surgery in two cases [10]. In our study, we did not observe any patient surgeries being converted to open surgery. No significant difference was observed between the group with stone disease and the group without stone disease with regard to the requirement for transfusion; furthermore, intraoperative bowel damage was observed in one patient in each of the two groups. 

Our study has some limitations. The main limitations of our study are the small population size and its retrospective nature. Another limitation is that stone analysis was not performed in the patients who had kidney stone after the nephrectomy. Despite the fact that all of the cases were operated by surgeons experienced in laparoscopic surgery, the fact that all cases were not operated by a single surgeon can be considered a limitation. In non-functional kidney patients, the effect of the presence of stone on the results of laparoscopic surgery and on morbidity may be better understood with a higher number of patients and further prospective multicentered studies.

## Conclusions

We observed that except for the postoperative complication rates, the laparoscopic nephrectomy results in patients with hydronephrotic non-functional kidneys with or without stone were similar. In the group with stones, dense adhesions secondary to previous infection may render the surgery difficult. We believe that in order to prevent serious postoperative complications, the kidney dissection should be performed more carefully and cautiously.
